# Supplementary material for: Impact of IBD-Associated Dysbiosis on Bacterial Quorum Sensing Mediated by Acyl-Homoserine Lactone in Human Gut Microbiota
Source: Int J Mol Sci. 2022 Dec 6;23(23):15404. doi: 10.3390/ijms232315404 (PMC9738069; doi:10.3390/ijms232315404)
Supplement: Supplementary file 1 [file ijms-23-15404-s001.zip › Figure S1. ROC curves and species specificity.pdf]

Supplementary Figure S1. ROC curves and species specificity

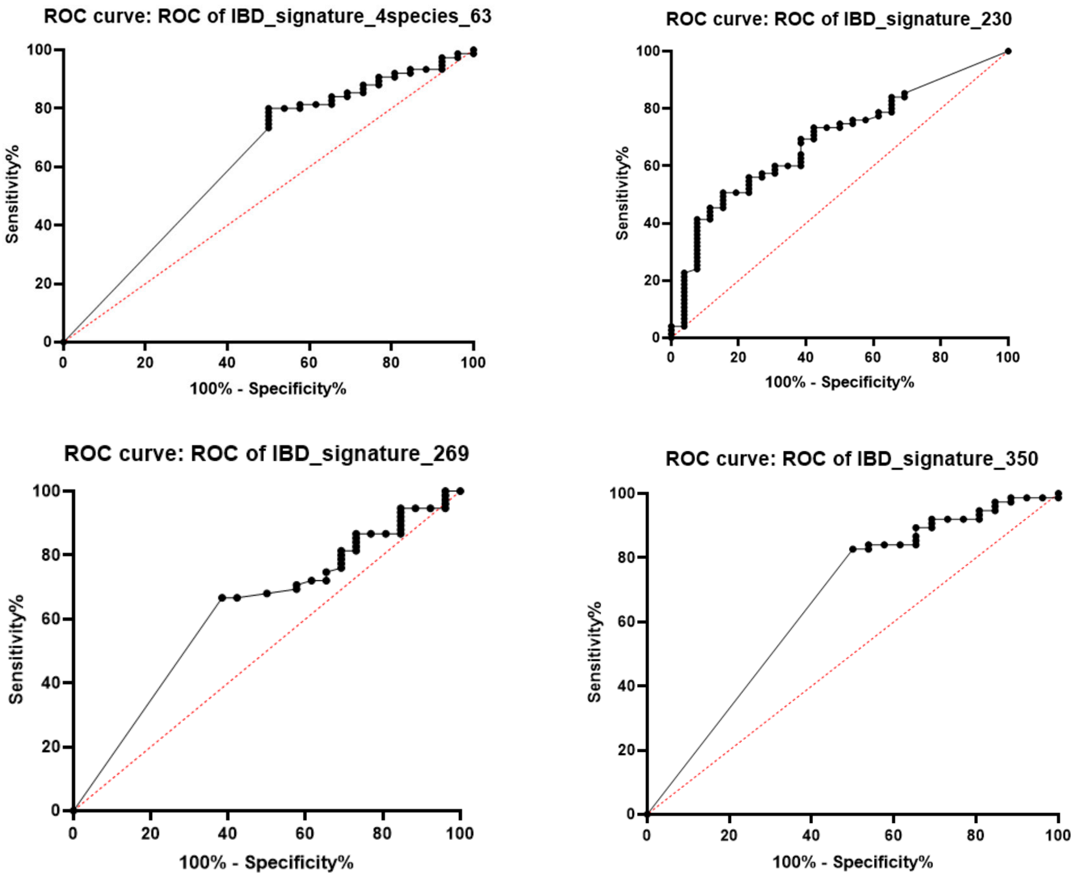

63: _Barnesiella intestinihominis

230: _Flavonifractor plautii

269: _Firmicutes_bacterium_CAG_83

350: _Akkermansia_muciniphila

Species	Threshold (Rarefied data)	Sensitivity	95 CI	Specificity%	95% CI	Likelihood ratio
63	< 1.500	73.33	62.37% to 82.02%	50.00	32.06% to 67,94%	1,467
230	> 1973	4.0	1,090% to 11,11%	100,0	87,13% to 100,0%	
269	< 1.500	66,67	55,42% to 76,29%	57,69	38,95% to 74,46%	1,576
350	< 0.5000	82,67	72,57% to 89,58%	50,00	32,06% to 67,94%	1,653
