# Supplementary material for: Impact of IBD-Associated Dysbiosis on Bacterial Quorum Sensing Mediated by Acyl-Homoserine Lactone in Human Gut Microbiota
Source: Int J Mol Sci. 2022 Dec 6;23(23):15404. doi: 10.3390/ijms232315404 (PMC9738069; doi:10.3390/ijms232315404)
Supplement: Supplementary file 1 [file ijms-23-15404-s001.zip › File S5. HPLC-MS protocol.pdf]

Supplementary File S5. AHL extraction and LC-MS/MS detection

AHL extraction

One gram of lyophilized faeces was divided into 8 tubes of 50 mL and 20 mL of 0.9% NaCl was added to each tube to solubilise it, then 20 mL of high-performance liquid chromatography (HPLC)-grade ethyl acetate + formic acid 0.1% (Carlo Erba) was added. Two µL of the internal standard N-hexanoyl-L-homoserine lactone-d3 (C6d3-HSL, Cayman Chemical) at the concentration of 2.5mM per tube was used as an intern standard. The tubes were shaken vigorously for 30 seconds to form an emulsion and allowed to stand several times. After centrifugation for 10 min at 3000 rpm, the organic phase was recovered (upper phase) and placed in a 1 litre flask. Again 20 ml of acetate + 0.1% formic acid was added to the aqueous phase. The tubes were shaken and centrifuged again, and the organic phases were pooled in the flask. The organic phase was evaporated using a rotatory evaporator and then taken up in 5 mL of acetonitrile diluted with 45 mL of water + 0.1% formic acid. The sample was then passed through a reverse-phase C18 cartridge (Sep-Pak Waters 12cc 2g). The column was washed with water several times and then washed with cyclohexane (10mL). The samples were eluted with 10 mL of methanol and evaporated under nitrogen. For mass spectrometry, they were taken up in 1ml of acetonitrile and then injected at 2 µL.

AHL detection with LC-MS/MS

Samples were analysed using an LC-20ADXR chromatographic system (Shimadzu, Kyoto, Japan) in tandem with a QTRAP 5500 quadrupole linear ion trap MS/MS spec-trometer system (SCIEX, Ontario, Canada). Chromatographic separation was performed with a kinetex column (100 x 2.1 mm; particle size 5 µm) (Phenomenex, Torrance, USA) with a 2.1 mm UHPLC C8 SecurityGuard™ ULTRA Cartridges column (Phenomenex, Torrance, USA). The mobile phases consisted of 0.1% formic acid (FA) in water (v/v) (mobile phase A) and 0.1% FA in acetonitrile (mobile phase B). The column temperature was set at 40 °C, MS detection was performed using electrospray ionisation (ESI) in positive mode using the Multiple Reaction Monitoring (MRM) function of the analyser. The linear gradient for AHL elution was programmed to last 23 minutes. The instrument parameters were set as follows: fog and turbo gas: 30 psi, curtain gas: 20 psi, ion spray voltage: 5,500 V for positive ionisation, source temperature: 400 °C. De-clustering potentials (DP) were set at 60 V. Spectral data acquisitions were processed using Analyst software (v1.6.3) in the multiple reaction monitoring mode. As described by Cataldi et al., identification of an AHL (designated by m/z of the precursor ion [M+H]+) was defined by the presence of the precursor ion and the two possible product ions ([M+H -101]+, neutral loss and 102 or lactone moiety) at the same retention time. The AHL was expressed in relative concentrations and normalisation relative to the internal standard (C6-d3-HSL). The quantifications were performed with Multiquant software (v3.0.2) (SCIEX, Ontario, Canada).
