# Supplementary material for: Impact of IBD-Associated Dysbiosis on Bacterial Quorum Sensing Mediated by Acyl-Homoserine Lactone in Human Gut Microbiota
Source: Int J Mol Sci. 2022 Dec 6;23(23):15404. doi: 10.3390/ijms232315404 (PMC9738069; doi:10.3390/ijms232315404)
Supplement: Supplementary file 1 [file ijms-23-15404-s001.zip › Table S1.Patientí»s characteristics for HPLC-MSMS detection .pdf]

**Supplementary Table S1. Patient's characteristics for HPLC-MS/MS detection**

| Group | Characteristics |
|---|---|
| **Non-IBD controls** | 10 healthy (non-IBD) controls with no digestive pathology in their history between 23 and 61 years old. One had been exposed to antibiotics in the 3 months prior to stool collection. Only one was an active smoker. |
| **IBD patients** | Patient A: 44-year-old male patient with ileocolic Crohn's disease with anoperineal location for 23 years, he had in a severe relapse of his disease with a localized abscess in the ileum being treated with intravenous antibiotics and exclusive enteral nutrition at the time of sampling. He had not been exposed to corticosteroids in the 3 months prior to the stool sample. Smoking had been stopped for over a year.<br><br>Patient B: 65-year-old male patient with ileal Crohn's disease diagnosed 34 years ago. He was hospitalised for a moderate relapse of his disease with therapeutic escape from anti-TNF-α drugs. He had been exposed to high-dose corticosteroid therapy for 3 months prior to sampling. He had not been exposed to antibiotics during the 3 months prior to sampling. Smoking had been stopped for over a year. |
