# Supplementary material for: Impact of IBD-Associated Dysbiosis on Bacterial Quorum Sensing Mediated by Acyl-Homoserine Lactone in Human Gut Microbiota
Source: Int J Mol Sci. 2022 Dec 6;23(23):15404. doi: 10.3390/ijms232315404 (PMC9738069; doi:10.3390/ijms232315404)
Supplement: Supplementary file 1 [file ijms-23-15404-s001.zip › Table S3. Microbiota composition analysis.pdf]

Supplementary Table S3. Microbiota composition analysis. * $p < 0.05$, ** $p < 0.005$.

Phyla	non-IBD	CD	UC	p-value Kruskal-Wallis
**Bacteroidetes**	71.1 +/- 5.0	72.7 +/- 3.8	66.8 +/- 4.27	0.28
**Firmicutes**	27.3 +/- 5.0	30.0 +/- 2.6	31.0 +/- 4.0	0.11
**Proteobacteria**	0.7 +/- 0.2	3.8 +/- 1.7	0.2 +/- 0.2	0.39
**Actinobacteria**	0.4 +/- 0.1	0.6 +/- 0.2	1.1 +/- 0.4	0.06
**Verrucomicrobia**	0.5 +/- 0.2	1.8 +/-1.9	0.2 +/- 0.2	** 0.0042

**Species** (Mean +/- SEM)	**non-IBD**	**CD**	**UC**	**p-value Kruskal-Wallis**
B. vulgatus	15.4 +/- 2.9	20.3 +/- 2.9	16.6 +/- 3.1	0.81
P. copri	11.0 +/- 4.8	5.1 +/- 2.5	5.3 +/- 3.2	0.44
F. prausnitzii	10.5 +/- 3.0	7.3 +/- 1.6	9.3 +/- 1.6	0.13
B. uniformis	8.6 +/- 1.8	11.3 +/- 1.8	11.0 +/- 2.1	0.68
B. dorei	5.5 +/- 2.6	4.3 +/- 1.8	4.5 +/- 1.7	0.84
B. stercoris	5.0 +/- 1.9	5.5 +/- 1.5	5.2 +/- 1.4	0.49
R. faecis	3.9 +/- 1.7	1.2 +/- 0.4	3.2 +/- 1.0	0.12
A. putredinis	3.7 +/- 0.7	2.8 +/- 0.5	1.8 +/- 0.5	*0.04
B. caccae	2.8 +/- 0.8	2.5 +/- 0.5	2.2 +/- 0.6	0.68
B. thetaiotaomicron	2.0 +/- 0.7	2.3 +/- 0.5	2.0 +/- 0.9	0.83
B. fragilis	1.5 +/- 1.1	2.7 +/- 1.1	2.7 +/- 1.7	0.43
A. muciniphila	0.5 +/- 0.2	1.8 +/- 1.5	0.2 +/- 0.2	**0.0042
E. coli	0.3 +/- 0.2	1.5 +/- 0.7	0.1 +/- 0.005	0.53
Pseudomonas sp.	0	0	$1.0\ 10^{-4}$ +/- $1\ 10^{-4}$	0.24
Campylobacter sp.	$2.3\ 10^{-3}$ +/- $2.3\ 10^{-3}$	$4.8\ 10^{-4}$ +/- $4.5\ 10^{-4}$	0	0.58
Klebsiella sp.	$5.0\ 10^{-3}$ +/- $3.7\ 10^{-3}$	$5.2\ 10^{-1}$ +/- $3.6\ 10^{-1}$	$2.1\ 10^{-3}$ +/- $1.9\ 10^{-3}$	0.53
Citrobacter sp.	$8.0\ 10^{-4}$ +/- $8.0\ 10^{-4}$	$1.5\ 10^{-2}$ +/- $1.5\ 10^{-2}$	0	0.58
***Enterobacter cloacae* complex**	0	0.8 +/- 0.8	0	0.11
