# Supplementary material for: Impact of IBD-Associated Dysbiosis on Bacterial Quorum Sensing Mediated by Acyl-Homoserine Lactone in Human Gut Microbiota
Source: Int J Mol Sci. 2022 Dec 6;23(23):15404. doi: 10.3390/ijms232315404 (PMC9738069; doi:10.3390/ijms232315404)
Supplement: Supplementary file 1 [file ijms-23-15404-s001.zip › Table S4. Gene list summarised.pdf]

Table S4. Gene list summarised

Function	QS system	Bacteria / Accession number	References
**Synthase**		603 bacteria	Doberva et al. 2015
**Receptor**	SdiA	*Escherichia coli* MG1655 NC_000913.3	Swearingen et al. 2008
	LasR	*Pseudomonas aeruginosa* PAO1 NC_002516.2	Le Berre et al. 2008
	RhlR	*Pseudomonas aeruginosa* PAO1 U40458.1	Brint et al. 1995
	EsaR	*Serratia marcescens* SmUNAM836	Sandner-Miranda et al. 2016
	CroR	*Citrobacter rodentium* ICC168	Petty et al. 2010
	LuxR	*Bacteroides fragilis* NCTC 9343	Pumbwe et al. 2008
	LuxR	*Bacteroides dorei* FYB91_RS16330	
