# Supplementary material for: Impact of IBD-Associated Dysbiosis on Bacterial Quorum Sensing Mediated by Acyl-Homoserine Lactone in Human Gut Microbiota
Source: Int J Mol Sci. 2022 Dec 6;23(23):15404. doi: 10.3390/ijms232315404 (PMC9738069; doi:10.3390/ijms232315404)
Supplement: Supplementary file 1 [file ijms-23-15404-s001.zip › Table S7. AHL receptor analysis according to phenotype.pdf]

Supplementary Table S7. Relative abundance and expression of AHL receptor genes according to disease phenotype

Relative abundance (p-value Mann-Whitney U test)				
**Genes**	**Non-IBD vs IBD**	**Non-IBD vs CD**	**Non-IBD vs UC**	**CD vs UC**
sdiA	0.67	0.32	0.48	0.10
luxR1 B. fragilis	0.18	0.12	0.52	0.37
luxR2 B. fragilis	0.40	0.22	0.99	0.26
luxR3 B. fragilis	0.23	0.19	0.48	0.61
luxR4 B. fragilis	0.36	0.25	0.77	0.50
luxR1 B. dorei	0.52	0.58	0.55	0.85
**Expression (p-value Mann-Whitney U test)**<br>*(n vs n) : number of patients in each subgroup with gene presence in their metagenome				
**Genes**	**Non-IBD vs IBD**	**Non-IBD vs CD**	**Non-IBD vs UC**	**CD vs UC**
sdiA	0.90<br>(8 vs 26)	0.95<br>(8 vs 20)	> 0.99<br>(8 vs 6)	> 0.99<br>(20 vs 6)
luxR1 B. fragilis	0.78<br>(8 vs 36)	0.75<br>(8 vs 25)	0.92<br>(8 vs 11)	0.60<br>(25 vs 11)
luxR2 B. fragilis	0.42<br>(10 vs 35)	0.38<br>(10 vs 25)	0.74<br>(10 vs 10)	0.79<br>(25 vs 10)
luxR3 B. fragilis	0.29<br>(8 vs 33)	0.50<br>(8 vs 22)	0.15<br>(8 vs 11)	0.18<br>(22 vs 11)
luxR4 B. fragilis	0.82<br>(10 vs 36)	0.77<br>(10 vs 25)	0.99<br>(10 vs 11)	0.97<br>(25 vs 11)
luxR1 B. dorei	* 0.0233<br>(24 vs 70)	* 0.0229<br>(24 vs 44)	0.11<br>(24 vs 26)	0.97<br>(44 vs 26)
