# Supplementary material for: Impact of IBD-Associated Dysbiosis on Bacterial Quorum Sensing Mediated by Acyl-Homoserine Lactone in Human Gut Microbiota
Source: Int J Mol Sci. 2022 Dec 6;23(23):15404. doi: 10.3390/ijms232315404 (PMC9738069; doi:10.3390/ijms232315404)
Supplement: Supplementary file 1 [file ijms-23-15404-s001.zip › Table S8. AHL receptor analysis according to dysbiosis.pdf]

Supplementary Table S8. Relative abundance and expression of AHL receptor genes according to dysbiosis

Relative abundance (p-value Mann-Whitney U test)				
**Genes**	**ND (n=54) vs D (n=47)**	**ND-nonIBD (n=20) vs D-IBD (n=41)**	**ND (n=54) vs D-IBD (n=41)**	**ND-IBD (n=34) vs D-IBD (n=41)**
sdiA	0.72	0.81	0.58	0.54
luxR1 B. fragilis	0.99	0.38	0.89	0.71
luxR2 B. fragilis	0.52	0.89	0.64	0.45
luxR3 B. fragilis	0.96	0.44	0.95	0.68
luxR4 B. fragilis	0.58	0.80	0.69	0.47
luxR1 B. dorei	0.76	0.79	0.99	0.87
**Expression (p-value Mann-Whitney U test)**<br>*(n vs n) : number of patients in each subgroup with gene presence in their metagenome				
**Genes**	**ND vs D**	**ND-nonIBD vs D-IBD**	**ND vs D-IBD**	**ND-IBD vs D-IBD**
sdiA	0.73<br>(17 vs 15)	0.77<br>(7 vs 14)	0.73<br>(17 vs 14)	0.92<br>(10 vs 14)
luxR1 B. fragilis	0.66<br>(24 vs 19)	0.56<br>(6 vs 17)	0.79<br>(24 vs 17)	> 0.99<br>(18 vs 17)
luxR2 B. fragilis	0.47<br>(27 vs 17)	0.92<br>(8 vs 15)	0.84<br>(27 vs 15)	0.84<br>(19 vs 15)
luxR3 B. fragilis	0.64<br>(22 vs 18)	0.17<br>(6 vs 16)	0.74<br>(22 vs 16)	0.81<br>(16 vs 16)
luxR4 B. fragilis	* 0.0383<br>(27 vs 18)	0.34<br>(8 vs 16)	* 0.04<br>(27 vs 16)	* 0.04<br>(19 vs 16)
luxR1 B. dorei	0.72<br>(52 vs 41)	0.14<br>(19 vs 36)	0.97<br>(52 vs 36)	0.34<br>(33 vs 36)

Groups:
ND: non-dysbiotic
D: dysbiotic
ND-nonIBD: nondysbiotic non-IBD
D-IBD: dysbiotic-IBD
ND-IBD: non-dysbiotic IBD
